# Reliability and validity of the Safe Routes to school parent and student surveys

**DOI:** 10.1186/1479-5868-8-56

**Published:** 2011-06-08

**Authors:** Noreen C McDonald, Amanda E Dwelley, Tabitha S Combs, Kelly R Evenson, Richard H Winters

**Affiliations:** 1 Department of City & Regional Planning, University of North Carolina at Chapel Hill, CB#3140, Chapel Hill, NC 27599, USA; 2 Department of Epidemiology, University of North Carolina at Chapel Hill, CB# 8050, Chapel Hill, NC 27514, USA; 3 Mecklenburg County Department of Public Health, 249 Billingsley Rd, Charlotte, NC 28211, USA

## Abstract

**Background:**

The purpose of this study is to assess the reliability and validity of the U.S. National Center for Safe Routes to School's in-class student travel tallies and written parent surveys. Over 65,000 tallies and 374,000 parent surveys have been completed, but no published studies have examined their measurement properties.

**Methods:**

Students and parents from two Charlotte, NC (USA) elementary schools participated. Tallies were conducted on two consecutive days using a hand-raising protocol; on day two students were also asked to recall the previous days' travel. The recall from day two was compared with day one to assess 24-hour test-retest reliability. Convergent validity was assessed by comparing parent-reports of students' travel mode with student-reports of travel mode. Two-week test-retest reliability of the parent survey was assessed by comparing within-parent responses. Reliability and validity were assessed using kappa statistics.

**Results:**

A total of 542 students participated in the in-class student travel tally reliability assessment and 262 parent-student dyads participated in the validity assessment. Reliability was high for travel to and from school (kappa > 0.8); convergent validity was lower but still high (kappa > 0.75). There were no differences by student grade level. Two-week test-retest reliability of the parent survey (n = 112) ranged from moderate to very high for objective questions on travel mode and travel times (kappa range: 0.62 - 0.97) but was substantially lower for subjective assessments of barriers to walking to school (kappa range: 0.31 - 0.76).

**Conclusions:**

The student in-class student travel tally exhibited high reliability and validity at all elementary grades. The parent survey had high reliability on questions related to student travel mode, but lower reliability for attitudinal questions identifying barriers to walking to school. Parent survey design should be improved so that responses clearly indicate issues that influence parental decision making in regards to their children's mode of travel to school.

## Introduction

Low levels of physical activity among youth and the rise in childhood overweight and obesity have focused attention on interventions to increase physical activity [1,2]. The federal Safe Routes to School (SRTS) program aims to increase rates of walking and biking to and from school by improving safety and environmental conditions around schools [3,4]. Students that walk or bicycle to school generally have higher overall physical activity levels [5,6] and some studies have found associations between walking and biking to school, body composition, and cardiorespiratory fitness [7].

A critical component of the SRTS program is the collection of school-level data on how children travel between home and school. This information is used to assess mobility, select schools for SRTS funding, monitor SRTS programs, and evaluate SRTS programs. The National Center for SRTS - the federal clearinghouse - developed standardized forms to collect data from students and parents on school travel [8]. Between August 2008 and July 2010, over 65,000 classrooms collected data on travel to and from school and over 374,000 parent surveys were completed. Despite the broad scope of school travel data collection, no published studies have examined the measurement properties of these instruments using the protocols established by the National Center for SRTS.

The goal of this study was to assess the test-retest reliability and convergent validity of the two survey instruments that are the national standard for collecting data on school travel and assessing parental attitudes toward walking and bicycling. The first instrument is an in-class student travel tally which uses a hand-raising protocol to collect data on student travel modes to and from school. The second survey instrument is a parent travel survey that is sent home to parents and returned to the teacher, both via the student. The two-page form asks about children's school travel and what influenced their decision to allow their child to walk or bicycle.

## Methods

### Population and sample

Students and parents in the Charlotte-Mecklenburg School District, in Charlotte, North Carolina, participated in this study in May and June of 2010. Two elementary schools were chosen based on their participation in SRTS programs and their socioeconomic diversity. At School A, 13% of students were classified as economically disadvantaged (eligible for free or reduced lunch), 77% of students were white, 13% were African-American, and 5% Hispanic. At School B, 85% of students were classified as economically disadvantaged, 5% of students were white, 66% were African-American, and 26% were Hispanic [9]. All students enrolled in kindergarten through grade 5 were eligible for the study, and all parents of students enrolled in kindergarten through grade 5 were recruited for parent travel survey testing. The University of North Carolina at Chapel Hill Institutional Review Board and the Charlotte-Mecklenburg Schools approved the study procedures.

### Procedures

The analysis assessed the test-retest reliability and convergent validity of two survey instruments developed by the National Center for SRTS. The first instrument, the student travel tally, measured student-report of mode to and from school through hand-raising [10] (see Additional File 1). The second instrument was a pen-and-paper survey of parents on issues related to walking and biking to school [11] (see Additional File 2).

#### Student Travel Tally: 24-hour Test-Retest Reliability

The National Center's student travel tally measured student-report of school travel modes through two questions: "How did you get to school today?" and "How will you get home after school today?". Students raised their hands in response to the six travel mode categories available on the survey instrument - walk, bicycle, school bus, family vehicle, carpool or other. Parent and teacher volunteers familiar with student names were instructed to record each child's response on a class roster. Training occurred prior to the start of the first survey day. To assess the test-retest reliability of the instrument, school volunteers administered the student travel tally to students in study classrooms on day one. The following day the survey was repeated, along with an additional recall survey in which students were asked "How did you get to school yesterday?" and "How did you get home from school yesterday?" Twenty-one of 29 classrooms in School A participated in both the test and the re-test. At School B, all 21 classrooms participated in the first test, and one classroom per grade participated in the re-test on the second day, for a total of 27 test-retest classrooms in the two schools. The final sample was composed of 542 students providing 24-hour test-retest reliability of mode to school, and 468 students providing test-retest reliability of mode home from school.

#### Student Travel Tally: Convergent Validity

Assessment of the validity of student-report of travel mode was made by comparison with parent-report. Student-reports of school travel mode were collected on two sequential days using the student travel tally questions described above. Parent participants were contacted on each day of the study by phone or email as indicated in their returned consent form. Parents were asked "How did [child's name] get to school today?" and "How did [child's name] get home after school today?" Parents that could not be reached on the same day as the student-report of school travel mode were contacted 24 and 48 hours later and asked to recall information for each day. Parent-report of student travel mode was collected in Spanish and English. There were 28 classrooms in school A and 19 classrooms in school B in the convergent validity assessment. A total of 262 parent-student dyads provided convergent validity information on 418 trips to school and 370 trips from school to home. Researchers provided classroom supplies as incentives.

#### Parent Survey: Two-Week Test-Retest Reliability

The National Center for SRTS's parent survey instrument asked for parent-report of the child's *usual *travel mode to and from school, travel time to and from school, grade at which the child may be allowed to walk or bicycle, and barriers to walking or biking to school. The survey was distributed to parents via students about ten days before the classrooms tallies were taken. Parents were asked to return the surveys to their children's teachers within one week. Follow-up surveys were mailed to all families that provided a mailing address on the initial survey approximately one week after teachers collected completed initial surveys. Parents at School B received English and Spanish language versions of the parent survey, and a Spanish follow-up survey if they returned a Spanish version initially. Parents were given a classroom incentive for returning both survey forms. Follow-up survey responses were matched to initial surveys by a household ID code, child's gender, and child's grade. 112 parents from 50 classrooms participated in initial and follow-up parent surveys.

### Analysis

The test-retest reliability and convergent validity of the student travel tally were assessed with percent agreement and un-weighted *kappa *statistics. Results were disaggregated by potential sources of variation including school, grade level, parent contact mode (i.e. phone vs. email), and day of parent recall (i.e., same day, 24 - 48 hours later). Convergent validity analyses did not account for potential correlation among parent-student dyads that provided two days of reports on school travel, but did test whether multiple responses affected results.

Percent agreement and kappa statistics were used to assess test-retest reliability of parent survey responses. For travel mode and attitudinal questions, an un-weighted kappa statistic with missing values treated as valid responses was used. Reliability of distance-related and Likert-scale questions were assessed with a weighted kappa statistic and with an un-weighted kappa statistic with missing values treated as valid responses [12]. Results were analyzed by potential sources of variation such as school and grade level.

All analyses were conducted in Stata (Version 11.1, College Station, TX) in 2010. Qualitative judgments of level of agreement were based on the scale used by Landis and Koch [13], in which a kappa between 0.6-0.8 is considered substantial agreement, and kappa between 0.8-1.0 is considered almost perfect agreement.

## Results

### Student Travel Tally: Test-Retest Reliability

Participation rates were high for the reliability test of the student travel travel tally with 542 students (88.6% of eligible students) providing data on the trip to school and 468 students (76.5% of eligible students) providing the data for the trip home from school. Students were distributed across grades (Table 1). This sample size was sufficient to provide 80% power, assuming a null hypothesis of kappa ≤ 0.4 and an observed kappa of 0.9 (as observed by Evenson, et al. (2)).

**Table 1:** Survey Demographics

	Test-Retest Reliability of Student Tally		Convergent Validity of Student Tally		Test-Retest Reliability of Parent Survey	
	To n (%)	From n (%)	To n (%)	From n (%)	To n (%)	From n (%)

**Sample Size**	542	468	418	370	112	112

**Gender**						

Male	n/a	n/a	n/a	n/a	63 (56)	

Female	n/a	n/a	n/a	n/a	49 (44)	

**Grade**						

Kindergarten	124 (23)	124 (27)	86 (21)	77 (21)	23 (21)	

1^st^	36 (7)	37 (8)	53 (13)	56 (15)	19 (17)	

2^nd^	53 (10)	34 (7)	60 (14)	53 (14)	16 (14)	

3^rd^	97 (18)	70 (15)	82 (20)	68 (18)	18 (16)	

4^th^	138 (25)	133 (28)	97 (23)	87 (24)	22 (20)	

5^th^	94 (17)	70 (15)	40 (10)	29 (8)	14 (12)	

**School**						

A	444 (82)	387 (83)	281 (67)	247 (67)	90 (80)	

B	98 (18)	81 (17)	137 (33)	123 (33)	22 (20)	

**Mode**						

Walk	18 (3)	32 (7)	16 (4)	27 (7)	10 (9)	8 (7)

Bicycle	4 (1)	3 (1)	7 (2)	6 (2)	1 (1)	2 (2)

School bus	184 (34)	296 (63)	160 (38)	244 (66)	56 (50)	76 (70)

Family vehicle	321 (59)	111 (24)	222 (53)	77 (21)	44 (39)	21 (19)

Carpool	13 (2)	7 (2)	13 (3)	8 (2)	1 (1)	1 (1)

Other	2 (0)	19 (4)	0 (0)	8 (2)	0 (0)	1 (1)

n/a = Not applicable because information not collected

As shown in Table 2, agreement between travel modes reported and recalled was near perfect. Table 3 shows that percent agreement and kappa statistics similarly showed almost perfect within each grade level. There was also no significant difference in agreement across schools based on kappa scores (p = 0.67).

**Table 2:** Test-Retest Reliability and Convergent Validity of the SRTS Student Travel Tally

	Six Travel Modes (surveyed)			Five Travel Modes (family vehicle & carpool combined)		
	n	Mean % Agreement	Mean Kappa (95% CI)	n	Mean % Agreement	Mean Kappa (95% CI)

**Test-Retest of Student Responses**						

Trip to school	542	92.8	0.86 (0.79, 0.93)	542	94.1	0.88 (0.81, 0.96)

Trip home	468	92.1	0.85 (0.78, 0.92)	468	92.9	0.86 (0.79, 0.93)

**Comparison of Student and Parent Responses**						

Trip to school	418	86.8	0.78 (0.70, 0.85)	418	90.4	0.82 (0.74, 0.91)

Trip home	370	88.1	0.77 (0.70, 0.84)	370	91.1	0.82 (0.75, 0.90)

CI = confidence interval

**Table 3:** Test-Retest Reliability of the SRTS Student Travel Tally by grade level of student

		Trip to School			Trip Home After School		
	Grade	n	Mean % Agreement	Mean Kappa (95% CI)	n	Mean % Agreement	Mean Kappa (95% CI)

**Student Test-Retest**							

	K	124	91.9	0.83 (0.67, 1.00)	124	94.4	0.90 (0.77, 1.03)

	1	36	100.0	1.00 (0.70, 1.30)	37	94.6	0.86 (0.61, 1.11)

	2	53	96.2	0.92 (0.66, 1.18)	34	85.3	0.71 (0.47, 0.95)

	3	97	90.7	0.85 (0.72, 0.99)	70	91.4	0.84 (0.68, 1.01)

	4	138	93.5	0.85 (0.71, 0.99)	133	94.7	0.90 (0.76, 1.03)

	5	94	90.4	0.83 (0.67, 0.99)	70	85.7	0.75 (0.59, 0.91)

**Parent-Student Agreement**							

	K	86	81.4	0.68 (0.51, 0.85)	77	84.4	0.65 (0.50, 0.80)

	1	53	83.0	0.71 (0.50, 0.92)	56	91.1	0.81 (0.63, 0.99)

	2	60	91.7	0.85 (0.65, 1.06)	53	81.1	0.67 (0.49, 0.85)

	3	82	85.4	0.77 (0.62, 0.91)	68	86.8	0.76 (0.59, 0.92)

	4	97	93.8	0.89 (0.72, 1.06)	87	93.1	0.87 (0.71, 1.02)

	5	40	82.5	0.67 (0.46, 0.88)	29	93.1	0.89 (0.68, 1.10)

When there were differences between Day 1 and Day 2 recall, they were due to disagreements between family vehicle and school bus, the two most popularly reported modes (59.0% of disagreement in mode to school was due to students reporting arriving at school in a family vehicle or bus on day one and recalling having used the other mode on day two). Disagreements on the trip home were also disproportionately related to family vehicle responses: 70.0% of disagreement arose from reporting a family vehicle either during the day 1 test or the day 2 recall, and reporting a different mode (walk, bicycle, bus, carpool, or other) the other time. Given the potential for misclassification of family vehicle versus carpool responses [14], agreement statistics were also calculated using a combined *car *category (family vehicle and carpool). Test-retest reliability was marginally higher when car trips were combined (see Table 2), though the difference between the two kappa scores was not statistically significant.

### Student Travel Tally: Parent-Student Convergent Validity

Researchers collected parent-report of student travel mode from parents of 262 students (24% of 1,076 enrolled students). Participation rates were slightly higher for School A, than School B. Parent and student data were matched for a total of 418 trips to school (representing 260 parent-student dyads) and 370 trips home (representing 252 parent-student dyads) (Table 2). Assuming an observed kappa statistic of 0.80 (2) and a null hypothesis of kappa ≤ 0.4, this sample size provides at least 80% power (12).

Parent and student travel mode responses achieved substantial agreement for trips to school and for trips home (see Table 2). Agreement was also high within each grade (see Table 3). Convergent validity was higher at School A, the school with fewer economically disadvantaged students, than for School B for trips to school, but the differences were not significant (p = 0.08 for trips to school and p = 0.33 for trips from school). There were also no statistically significant differences in parent-student agreement by parent contact mode, i.e. phone vs. email, (p = 0.19) or by day of parent recall, i.e. same day vs. 24-48 hours later, (p = 0.55). Analyses with only one observation per parent-student dyad also found similar levels of agreement.

Parents reported that 5.8% of students walked or bicycled to school on study days, and the same proportion walked or bicycled home. The most common disagreements between parent and student report on the trip to school occurred when a student reported taking a family vehicle to school while the parent reported a carpool or a bus (representing a combined 47.3% of the disagreement). The most common disagreement on the trip home after school occurred when a student reported taking a family vehicle to school while the parent reported a carpool (18.2% of the disagreement), when a student reported a family vehicle and the parent reported walking, riding the bus, or 'other' (15.9% of the disagreement), or when a student reported walking and the parent reported a carpool (9.1% of the disagreement). Combining family vehicle and carpool into a single category yielded slightly better agreement (see Table 2).

### Parent Survey: Test-Retest Reliability

Across both schools, 427 initial parent surveys were returned (40% of students). One follow-up survey was mailed to each of the 343 participating household that provided a valid mailing address. Of these, 123 follow-up surveys were returned via mail (representing 11% of students). Eleven of these surveys were unable to be matched with children in the initial survey, leaving us with a final sample of 112 matched pairs of surveys. The final sample contains four households that returned Spanish language versions of the surveys. Response rates were lower in School B (7% of students) compared to School A (14% of students). Parents who returned both initial and follow-up surveys reported that 9.8% of children usually walked or bicycled to school, and 9.2% usually walked or bicycled home.

Parent surveys achieved substantial test-retest reliability on fact-based questions such as number of children in kindergarten through eighth grade, usual mode to school, and usual mode from school to home (see Table 4). Test-retest reliability was lower for ordinal-scale questions such as distance from child's home to school, length of time to get to school, and length of time from school to home. Two of the three Likert-scale questions showed only moderate agreement using weighted kappa (Q12-Q14 in Table 4). Test-retest agreement on grade at which child would be allowed to walk or bicycle was moderate.

**Table 4:** Test-Retest Reliability of the SRTS Parent Survey

	n	% Agreement	Kappa Coefficient (95% CI)
Q3: How many children do you have in Kindergarten through 8^th ^grade?	112	91.1	0.85 (0.72, 0.99)

Q5: How far does your child live from school?	112	72.3	0.65 (0.56, 0.74)

Q5: How far does your child live from school?	110	93.3	0.77 (0.65, 0.90) w

Q6a: On most days, how does your child arrive for school?	112	98.2	0.97 (0.83, 1.11)

Q6b: On most days, how does your child leave from school?	112	92.9	0.85 (0.72, 0.98)

Q7a: How long does it normally take your child to get to school?	112	75.9	0.67 (0.56, 0.77)

Q7a: How long does it normally take your child to get to school?	111	94.6	0.76 (0.64, 0.88) w

Q7b: How long does it normally take your child to get from school?	112	71.4	0.62 (0.52, 0.72)

Q7b: How long does it normally take your child to get from school?	108	94.3	0.76 (0.64, 0.88) w

Q8: Has your child asked you for permission to walk or bike to/from school in the past year?	112	86.6	0.74 (0.56, 0.92)

Q9: At what grade would you allow your child to walk or bike to/from school without an adult?	112	74.1	0.57 (0.48, 0.66)

Q9 alt: Would you allow your child to walk/bike at any grade?	112	87.5	0.74 (0.56, 0.92)

Q11a: My child already walks/bikes to school	112	96.4	0.76 (0.57, 0.94)

Q12: In your opinion, how much does your child's school encourage or discourage walking and biking to/from school?	112	68.8	0.47 (0.34, 0.59)

Q13: How much fun is walking or biking to/from school for your child?	112	72.3	0.61 (0.50, 0.72)

Q14: How healthy is walking or biking to/from school for your child?	112	72.3	0.51 (0.38, 0. 65)

Q15: What is the highest grade or year of school you completed?	112	96.4	0.93 (0.81, 1.04)

"w" specifies "weights 1-|i-j|/(k-1), where i and j index the rows and columns of the ratings by the two raters and k is the maximum number of possible ratings" (http://www.stata.com/help.cgi?kappa#Options)

Missing is coded as a valid value except for weighted kappa coefficients

Two questions assessed attitudinal factors influencing parents' decision to allow, or not allow, their child to walk or bicycle to/from school. The survey listed 12 possible factors and parents were asked to check all that "affected" their school travel decision and also indicate whether "changing or improving" each of the 12 factors would make it more likely the parent would let their child walk or bicycle to/from school. The reliability of both of these questions was generally low with kappa coefficients ranging from 0.31 to 0.76 for the 12 factors (Table 5).

**Table 5:** Test-Retest Reliability of the SRTS Parent Survey on barriers

	*Q10: What of the following issues affected your decision to allow, or not allow, your child to walk or bike to/from school? *(n = 112)		*Q11: Would you probably let your child walk or bike to/from school if this problem were changed or improved? *(n = 112)	
	% Agreement	Kappa Coefficient (95% CI)	% Agreement	Kappa Coefficient (95% CI)

Distance	78.6	0.57 (0.38, 0.75)	64.3	0.49 (0.37, 0.60)

Convenience	93.8	0.76 (0.58, 0.95)	65.2	0.35 (0.23, 0.47)

Time	79.5	0.46 (0.27, 0.64)	58.0	0.31 (0.19, 0.43)

After-school activities	83.9	0.43 (0.25, 0.61)	63.4	0.31 (0.19, 0.43)

Speed of traffic	67.9	0.36 (0.18, 0.54)	56.3	0.39 (0.27, 0.50)

Amount of traffic	71.4	0.44 (0.26, 0.62)	63.4	0.47 (0.35, 0.58)

Presence of adults to walk with	77.7	0.40 (0.21, 0.58)	63.4	0.37 (0.24, 0.49)

Sidewalks	80.4	0.54 (0.36, 0.73)	63.4	0.41 (0.28, 0.53)

Safety	67.0	0.34 (0.16, 0.52)	57.1	0.37 (0.26, 0.49)

Presence of crossing guards	77.7	0.31 (0.13, 0.49)	61.6	0.32 (0.20, 0.45)

Violence	84.8	0.68 (0.49, 0.86)	66.1	0.48 (0.36, 0.59)

Weather	82.1	0.60 (0.42, 0.79)	63.4	0.41 (0.30, 0.53)

Q10: Possible responses were Yes and No Response/Missing

Q11: Possible responses were Yes, No, Not Sure, and No Response/Missing

In both questions, 'missing' was a valid response for agreement analysis; separate analyses with missing values excluded (not shown) produced similar results

## Discussion

This study is the first to test the reliability and convergent validity of the National Center for SRTS survey instruments at each grade level using the hand-raising protocols recommended by Center. Student test-retest reliability and parent-student convergent validity of student-report of school travel have been tested in smaller samples [15], with a written student survey form [14,15], and with a small time gap between student test-retest [14]. Forman et al [16] assessed the reliability of parental attitudes to walking to school, but the National Center's parent survey has not been evaluated.

Test-retest reliability for student-report of school travel mode using hand-raising was high, with 93% and 92% agreement and kappa statistics of 0.86 and 0.85 for trips to and from school, respectively. This agreement was slightly lower than that reported by Evenson et al [15], who found 96% - 100% agreement and kappa scores of 0.88 - 1.00 between student reports of travel modes to and from school, and Mendoza et al [14], who found 98% agreement and a kappa score of 0.97 between student reports of travel modes to school. Both of those studies used pen and paper to collect student-reports of school travel mode rather than hand-raising.

Parent-student convergent validity of school travel mode was high, with 87 - 88% agreement and kappa statistics of 0.77 - 0.78 for trips to and from school. Convergent validity was in line with that reported by Evenson et al [15] who reported 88 - 89% agreement and kappa statistics of 0.80 - 0.81, and Mendoza et al [14], who reported kappa statistics between 0.57 - 0.87 depending on how travel modes were categorized.

The use of hand-raising to collect school travel data displayed reasonable levels of reliability and convergent validity even in the youngest respondents. Kappa statistics for reliability and parent-student convergent validity for trips to or from school were similar across kindergarten through fifth grade, suggesting that young children are able to reliably report school travel mode.

Student test-retest reliability and convergent validity for the student travel tally were similar for trips to school and trips home. One might expect lower agreement for reports of the trip home, as students were asked to *predict *the afternoon trip on the first day, while on the second day students recalled a trip that had already happened. The similar percentage agreement and kappa for trips to and from school indicates that predicting trips home in advance of the trip was not a major source of disagreement. One might also expect more disagreement on trips to home after school if students stopped at intermediate destinations such as daycare or a friend's house, as reported by Evenson et al [15]. While the patterns of disagreement were slightly different for trips to school and home from school (Tables 3, 4), it was difficult to determine whether travel to intermediate destinations was a source of disagreement.

Researchers observed some confusion among parents, students, and volunteer survey administrators about the definitions of family vehicle, carpool, and other. For example, a student reporting a trip in a family vehicle and a parent reporting a carpool trip was one of the most common disagreements for trips to or from school. This confusion has been noted by other authors [14]. Calculating reliability and validity for five travel modes, with family vehicle and carpool combined, yields higher agreement and kappa statistics for both student test-retest and parent-student agreement (Table 2). Survey designers should consider combining family vehicle and carpool into one option.

Parent surveys showed substantial reliability for travel mode, travel time, education, and income questions. Test-retest reliability was unacceptably low for the subjective attitudinal questions, including grade level at which parents would allow children to walk or bicycle and barriers to walking and biking. Forman et al. [16] developed a similar parent survey instrument, in which they asked parents to rate the importance of seventeen different attitudinal factors on their decisions to allow their children to walk or bicycle to three types of destinations, including schools. Seven of their items roughly paralleled attitudinal questions on the SRTS parent survey, but had remarkably higher reliability (ICC ranging from 0.6 to 0.8). The difference in reliability might be explained by their use of a 5-point Likert scale, rather than the yes/no check boxes used in the SRTS parent survey. Survey designers should consider restructuring the questions on barriers to walking and biking to school with simplified questions and Likert-scale responses.

### Limitations

Limitations of this study include the relatively low proportion of walking and biking in the sample, variation in student survey administration methods by classroom, variation in parental contact method by school, and a relatively low response rate to parent surveys in the more socioeconomically disadvantaged school. Between 4 and 10% of students in the sample walked or bicycled to or from school in this study. If there are differences in reliability and validity by usual travel mode, then our results mainly reflect motorized travellers. Future research should assess variability by mode. Also, in-class tallies were administered by volunteers, and while all volunteers participated in a brief pre-survey training session, they may not have administered the surveys in exactly the same way. Also, volunteer survey administrators in some classrooms failed to collect data on return trips home (explaining the lower sample size for mode to home in both the 24-hour test-retest reliability assessment and the parent-student convergent validity assessment). Furthermore, some volunteers returned from their classrooms with anecdotes of students experiencing peer pressure to raise their hands inappropriately. However, this may be a more realistic test of what may actually happen in a classroom being surveyed.

## Conclusions

The student travel tally showed high test-retest reliability, and substantial convergent validity with parental responses. Agreement between student test-retest and parent-student responses was similar across kindergarten through fifth grade, suggesting that the student survey is appropriate across a wide range of ages. Some disagreements between parent and student responses were related to classification of travel modes, such as carpool and family vehicle. The parent survey showed high reliability for more objective questions, yet revealed variations in response patterns to barriers and attitudinal questions that suggest difficulty interpreting these questions. This information can be used to improve the design and language of attitudinal questions in future versions of school travel surveys and to facilitate analysis of data collected using the National Center for SRTS's survey instruments.

## Competing interests

The authors declare that they have no competing interests.

## Authors' contributions

NM designed the study, oversaw the data analysis, and developed the manuscript. AD led data analysis and interpretation and helped with data collection and manuscript development. TC developed the data collection protocol, supervised data collection, and assisted with data analysis and manuscript development. KE helped with study design and manuscript review and provided valuable input on data analysis. DW helped with school recruitment, data collection, and manuscript review. All authors have read and approved the final manuscript.
